# Mitochondrial growth during the cell cycle of *Trypanosoma brucei* bloodstream forms

**DOI:** 10.1038/srep36565

**Published:** 2016-11-22

**Authors:** Martin Jakob, Anneliese Hoffmann, Simona Amodeo, Camille Peitsch, Benoît Zuber, Torsten Ochsenreiter

**Affiliations:** 1 Institute of Cell Biology, University of Bern, Switzerland; 2 Graduate School for Cellular and Biomedical Sciences, University of Bern, Switzerland; 3 Institute of Anatomy, University of Bern, Switzerland

## Abstract

Mitochondrial organelles need to be replicated during cell division. Many aspects of this process have been studied in great detail, however the actual size increase and the position of organelle growth are less well understood. We use the protozoan parasite *Trypanosoma brucei* that contains a single mitochondrion to study organelle biogenesis by fluorescence microscopy. From the analysis of more than 1000 *T. brucei* bloodstream form cells of a nonsynchronous population we conclude that the mitochondrial network mostly grows from two areas along the main organelle axis, posterior and anterior of the nucleus. Loops and branches from these two areas eventually fuse to build a complex network. Together with the appearance of the division fold in the posterior part of the cell, pruning of the mitochondrial network and finally separation into the two daughter cells occurs. Overall organelle biogenesis is not continuous during cell growth and occurs mostly in the last part of the cell cycle. Furthermore, using 3D STED super resolution microscopy we reconstruct the volume of the organelle and characterize the region where the mitochondrial genome is positioned by serial block face scanning electron microscopy.

Mitochondria are essential organelles in eukaryotes for energy generation through oxidative phosphorylation. Aside from their function as the “power house” of the cell they are also involved in signalling, cell death and cell proliferation^1^. Mitochondria contain their own genome as well as their own transcription-, replication- and translation machinery^2^,^3^,^4^,^5^,^6^. While the vast majority of mitochondrial proteins are nuclear encoded, translated on cytoplasmic ribosomes and then imported into the organelle^7^, a few essential components of the respiratory chain, ribosomal proteins as well as ribosomal RNAs are encoded on most mitochondrial genomes^3^. During the cell cycle the mitochondrial organelles including their genomes have to be replicated and segregated to ensure faithful propagation to the daughter cell. In many organisms mitochondria display a dynamic behaviour of constant fission and fusion that responds to the energy demand of the cell^8^ and are involved in developmental and regulatory processes including apoptosis^9^. Key factors for the membrane fusion events are large GTPases in the outer and inner mitochondrial membrane (OM, IM)^10^,^11^,^12^. The master regulator of mitochondrial division is the soluble dynamin-related protein Dnm1 in yeast (Drp1 in mammals). Dnm1 is recruited and stimulated to oligomerise by Mdv1, which is bound to the OM via the membrane protein Fis1^8^.

Recent studies have identified endoplasmic reticulum (ER) mitochondrial contact sites as hot spots for mitochondrial division^13^,^14^. The ER mitochondrial encounter structure (ERMES) seems responsible for these contact sites and might also be involved in lipid exchange between the two organelles^15^.

Mitochondrial growth depends on the proper coordination of nuclear and mitochondrial gene expression^16^,^17^,^18^. Furthermore, correct import of the nuclear encoded proteins into the different sub-compartments of the organelle is essential. While there is a wealth of data on the components and mechanism of the protein import machinery^7^,^19^,^20^, the actual growth/mass increase of the organelle is not well understood. Early studies in human HeLa cells, green algae and the yeast *Candida albicans* showed that mitochondrial mass increase occurs continuously during the cell cycle and is well correlated with the increase in cell volume^21^,^22^,^23^. In human HeLa and *C. albicans* cells the mitochondrial volume makes up about 10% of the total cell volume at any time during the cell cycle, while in *Chlorella* this value is much lower (3%) but also constant throughout cell division^22^.

*T. brucei* belongs to a group of protozoa that only contain one mitochondrial organelle with one mitochondrial nucleoid. The nucleoid consists of 25 identical maxicircles encoding the genes for the respiratory chain, two ribosomal RNAs and a ribosomal protein as well as about 5000 minicircles encoding guide RNAs required for RNA editing of the mitochondrial mRNA transcripts^24^,^25^,^26^. Shape and size of the organelle varies depending on the life cycle stage of the parasite^25^,^27^. In the bloodstream form (BSF) the mitochondrial organelle lacks cristae and most of the complexes responsible for oxidative phosphorylation^27^ and thus does not produce energy. In this life cycle stage the organelle is a single tube stretching throughout the cell body from anterior to posterior, potentially replicating via several loop- and branch structures that are finally separated during cytokinesis^28^. In the insect form of the parasite the mitochondrial inner membrane is strongly enlarged to form cristae harbouring the oxidative phosphorylation complexes for energy production. Furthermore, the entire organelle is enlarged and now forms a complex network throughout the cell^27^. The *T. brucei* genome encodes a single dynamin-like protein TbDLP, which is involved in organelle division as well as endocytosis^29^. However, no fission-fusion dynamics have been demonstrated in trypanosomes although they require at least one fission event prior to cytokinesis. Also experiments inducing fission through the expression of the human Bax protein in insect form mitochondria demonstrate that once the mitochondrial network is vesiculated it can fuse to build a singular network when Bax is no longer expressed^31^. Since the parasite cells only contain a single mitochondrion its biogenesis is expected to be tightly integrated in the cell cycle^32^. The replication of the mitochondrial genome (kinetoplast DNA or kDNA) is initiated in G1 prior to nuclear replication, while the cells contain one kinetoplast and one nucleus (1K1N)^33^. During the nuclear S-phase the replicated mitochondrial genomes are segregated leading to cells with two kinetoplasts and one nucleus (2K1N). Driving force of the kDNA segregation are the basal bodies that are connected to the kDNA via the tripartite attachment complex (TAC)^34^. After mitosis and just prior to cytokinesis the cells contain two kinetoplasts and two nuclei (2K2N)^33^.

Here we describe mitochondrial growth and morphology during the cell cycle of *T. brucei* BSF and characterize the region around the kinetoplast DNA using quantitative imaging approaches including super resolution microscopy and serial block face scanning electron microscopy (SBFSEM).

## Materials and Methods

### Cell culture

BSF *T. brucei* (NYsm; New York Single marker^35^) were cultured at 37 °C and 5% CO_2_ in HMI-9 medium containing 10% fetal bovine serum.

### Fluorescence light microscopy

10^6^ cells were spread on slides, air-dried, and fixed with 4% PFA in PBS for 4 min. Cells were then permeabilised for 5 min with PBS containing 0.2% Triton-X 100. After each treatment the cells were washed with PBS 0.1% Tween-20. Blocking was achieved with PBS containing 4% BSA for 30 min. Primary and secondary antibodies were added for 1 h each at room temperature. After the second antibody incubation, cells were additionally washed with PBS. The cells were then covered by a film of PBS and HCS CellMask Green (ThermoFisher Scientific). Cells were mounted with ProLong^®^ Gold Antifade Mountant with DAPI (ThermoFisher Scientific). Primary and secondary antibodies were diluted in PBS containing 4% BSA as follows: anti-mtHSP70 (mouse, 1:4000)^36^ and Alexa Fluor^®^ 594 goat anti-mouse IgG (H + L) (1:1000, ThermoFisher Scientific). Images were acquired with a fluorescent microscope (Leica DM5500 B, 100x oil immersion phase contrast objective). Image analyses were performed in ImageJ.

### Stimulated emission depletion (STED) microscopy

For stimulated emission depletion (STED) microscopy^37^, cells were fixed on a slide with 4% PFA in PBS, permeabilised for 5 min with 0.2% TritonX-100 in PBS and blocked for 30 min with 4% BSA in PBS. As a primary antibody, mouse anti-mtHSP70 (1:4000)^36^ and as a secondary antibody Alexa Fluor^®^ 594 Goat-antiMouse IgG (H + L) (1:1000, ThermoFisher Scientific) in 4% BSA PBS were used. Cells were mounted with ProLong^®^ Gold Antifade Mountant with DAPI (Thermo Fisher Scientific). The images were acquired with a 100x objective on a Leica SP8 Confocal Microscope System with STED and de-convolved with Huygens professional software.

### Image enhancement–cell cycle montages

Montages were assembled in Adobe Photoshop. Single cells were cropped from the original images using a polygon selection. In order to align the cells in a posterior-anterior alignment, the cropped cells were tilted and/or mirrored. In order to emphasise the signal, median filters were applied and the local contrasts was enhanced using contrast limited adaptive histogramme equalisation (CLAHE). Linear brightness and contrast were adjusted.

### Feature extraction

Image hyperstacks were projected along the z-axis. Single cell segmentation was done using image tessellations based on local intensity maxima or Voronoi tessellation. The particles within the tessellation boundaries were then filtered according to their size and other properties. Topological skeletons of the mitochondria were assigned by retracing the course of the tubes manually from the pre-processed images. For each mitochondrion a main branch was determined, which was defined by the longest-shortest-path of the most parallel branch to the centerline of the cell body having its endpoints in close proximity to the endpoints of the pruned cell body.

### Serial block face scanning electron microscopy (SBFSEM)

Blocks staining, dehydration and embedding were performed as previously described^38^ with following modification: post-fixation was done in 0.15 M cacodylate buffer, 1.5% potassium ferrocyanide and 2% osmium tetroxide. Sample blocks were mounted on aluminum specimen pins (Gatan, Pleasonton, CA, USA) with a conductive silver epoxy glue. After a minimum drying time of 4 hours the blocks were imaged at the SBFSEM using a Quanta FEG 250 (FEI, Eindhoven, The Netherlands), equipped with a Gatan 3View2XP *in-situ* ultramicrotome (Gatan, Pleasonton, CA, USA). Pixel size was set to 6 nm and the slice thickness was set to 50 nm. Data processing, measurements and analysis were done using IMOD^39^. The kinetoplast area of the mitochondrion was boxed and moved into plane with the basal bodies by rotating the SFBSEM stack in three dimensions.

## Results and Discussion

### Automatic detection of kDNA and nuclear DNA

**Figure 1:**
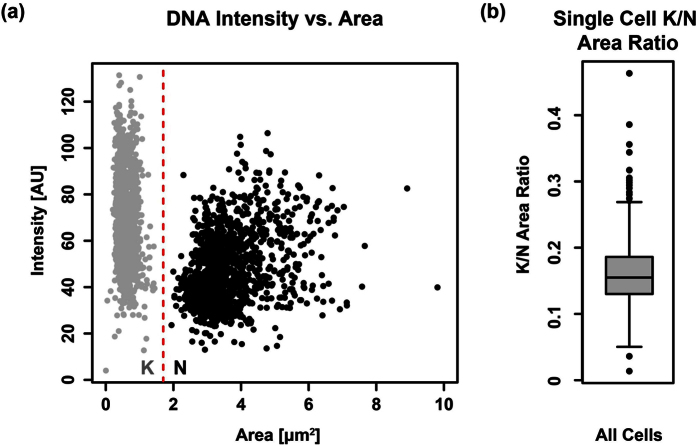
Area of the kDNA and the nuclei. (**a**) Scatter plot of average fluorescence intensity and area size of the DNA organelles. The red line defines the separation value that distinguishes kDNA and nuclei by area. (**b**) Boxplot showing the K/N Area Ratio on a single cell basis.

In this study we aimed to analyse how mitochondrial organelle growth is integrated in the cell cycle of *T. brucei*. We imaged 1381 bloodstream form (BSF) cells stained for (i) DNA by DAPI (ii) the mitochondrion using an antibody against the mitochondrial matrix protein mtHSP70 and (iii) the cell body using HCS CellMask, a dye staining the whole cell (Fig. S1). From each microscopic image stack individual cells were identified and features based on the area/intensity were measured (Fig. 1a). To determine the cell cycle stage we used the replication stage of the kDNA and the nuclear DNA. Both DNA particles were separated based on size (Fig. 1a). Since the kDNA particles never exceeded 50% of the nuclear size and the median ratio of kNDA/nucleus was below 0.2 (Fig. 1b) we considered any DNA particle smaller than 1.7 μm^2^ to be kDNA and any larger one to be a nucleus (red line in Fig. 1a).

### Definition of five distinct cell cycle stages

**Figure 2:**
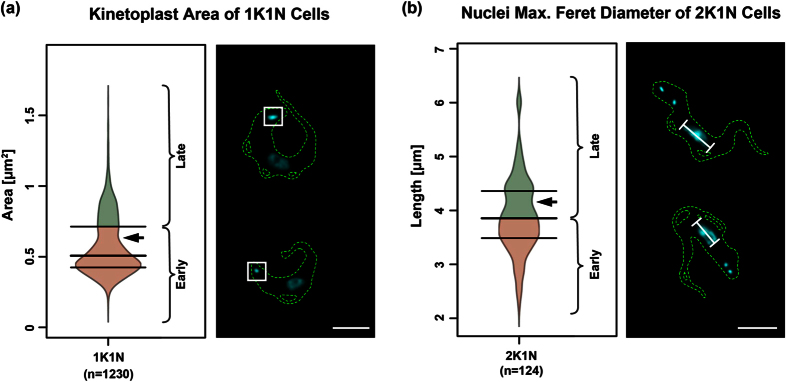
Clustering of 1K1N and 2K1N cells into “early” and “late”. The three horizontal lines on the bean plots depict the quartile-separators. The arrow heads point to the observed notch indicating bimodality. The lower part of the bean plots (red) show the cells assigned to the “early” stages and the upper part (green) show the “late” stages. Examples of cells stained with DAPI (cyan) from each cluster are shown on the right-hand side of the plots. The white squares in (**a**) indicate the area of the kDNA, the white bars in (**b**) indicate the longest axis (maximal feret diameter) of the nucleus. (**a**) Subdividing of 1K1N cells according to the area of the kinetoplast. (**b**) Subdivision of 2K1N cells according to the maximal feret diameter of the nuclei. Scale bar 5 μm.

In order to refine the cell cycle stages we used binary shape descriptors of the DNA organelles, the mitochondrion and the cell body. The descriptors that displayed the strongest bimodal characteristics, indicating distinct subpopulations, were chosen to define separation values for early and late replication stages of the respective organelle (Fig. 2). The separation values were defined by the quartile closest to the bimodal characteristic (arrows in Fig. 2). In the case of 1K1N cells, the area of the kinetoplast was used to distinguish an early and a late stage. The cut-off was chosen at its 75%-quartile (Fig. 2a). For 2K1N cells, the median of the longest axis (maximum feret diameter) of the nucleus was chosen to distinguish the early and late stage (Fig. 2b). These measurements allowed us to subdivide the 1381 randomly chosen cells into 901 early 1K1N, 296 late 1K1N, 59 early 2K1N, 60 late 2K1N and 65 2K2N cells corresponding to ~86% 1K1N, 9% 2K1N and 5% 2K2N cells and thus representing roughly an exponentially growing BSF population^40^.

### Measurement of the mitochondrial network and cell body size including area and length

**Figure 3:**
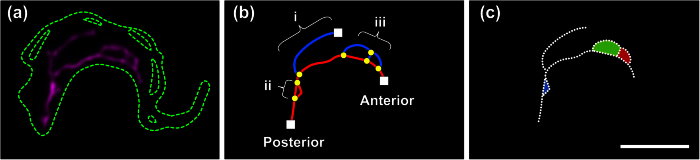
Schematic representation of a skeletonised mitochondrial network. (**a**) The mitochondrion (magenta) is shown as acquired by epifluorescence microscopy. The cell outlines as detected by CellMask are depicted by the dashed, green line. (**b**) A topological skeleton was extracted from (**a**). The mitochondrial main branch is depicted in red and the outgrowths are shown in blue. The white squares indicate endpoints, and the yellow circles indicate junctions. Outgrowth can be assigned into three major types: Shown here are (i) isolated branches, (ii) isolated loops, and (iii) complex structures, which can either be nested loops (multiple loops without endpoints) or complexes containing both, loops and branches (one or more loops with one or more endpoints). (**c**) Loops (blue, red, and green) are treated as shapes and can be measured accordingly. Scale bar 5 μm.

**Figure 4:**
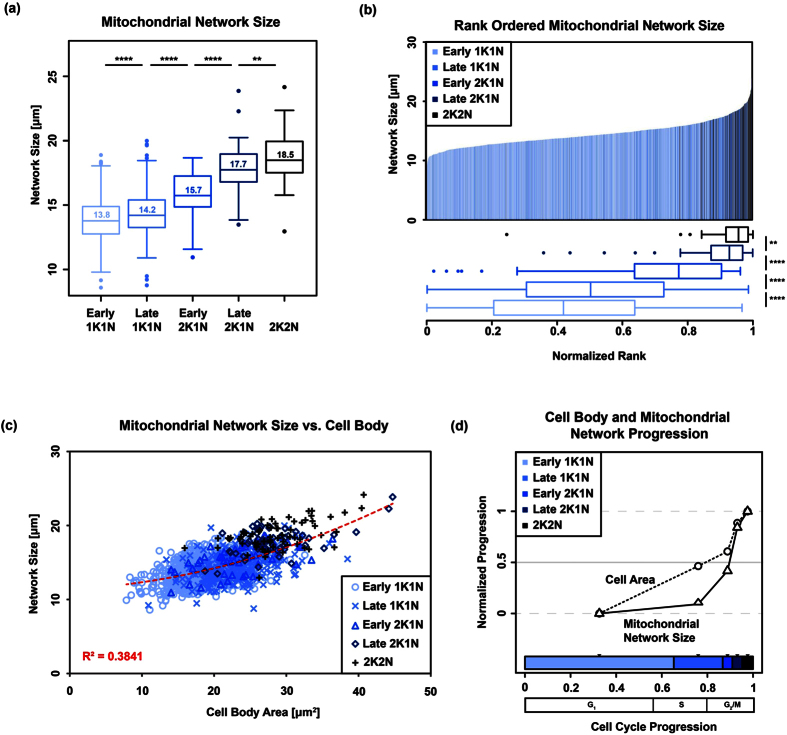
Mitochondrial network size during the cell cycle. The refined cell cycle stages are depicted in different colours, gradually changing from light blue (early 1K1N) to black (2K2N). (**a**) Boxplots of the total measured length of the mitochondrial skeleton for each refined cell cycle stage. The displayed numbers inside the boxes represent the median values. (**b**) The upper part shows a ranked statistic, each bin represents the mitochondrion of a single cell. The rank for each mitochondrion was normalised such that the shortest mitochondrion was assigned the value is 0 and the longest 1. The lower part depicts boxplots of the normalised ranks for each stage. Wilcoxon rank-signed tests were performed from one to the subsequent cell cycle stage. (**c**) Scatter plot of the cell body area against the total length of the mitochondrial skeleton. Each symbol on the plot represents a single cell. The cells from the refined cell cycle stages were grouped and are depicted in distinct colours and with distinct symbols. A quadratic regression coefficient (R^2^) for the overall data is shown. (**d**) Normalised median-values for cell area and mitochondrial network skeleton for the refined cell cycle stages on the y-axis. The values were normalised such that the lowest median value for each of the both measured properties is 0 and the highest is 1. The distances on the x-axis represent the cell cycle progression. The stacked, horizontal bar in the lower part of the plot shows the estimated duration of each cell cycle stage. The midpoints of the calculated cell cycle stages were chosen to place the median values on the y-axis. (****p-value < 0.0001, **0.001 < p-value < 0.01). We also added an estimation of the more conventional cell cycle stage nomenclature (G1, S, G2/M).

**Figure 5:**
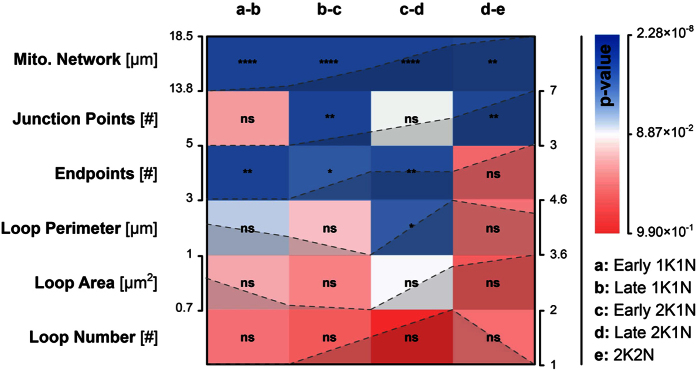
Heatmap matrix of mitochondrial growth during the cell cycle. Features including mitochondrial network size and number of endpoints were tested for significant changes from one cell cycle stage to the next (2-tailed unpaired student’s t-test). Each row represents one feature and each column represents two consecutive cell cycle stages (**a–e**). The colour code and the asterisks (****p-value < 0.0001, ***0.0001 < p-value < 0.001, **0.001 < p-value < 0.01, * = 0.01 < p-value < 0.05, ns = p-value ≥ 0.05) represent the obtained p-value and the significance level respectively. The tested features were arranged in descending order according to their average p-value. The dashed lines in the matrix show the trend of the calculated median values for each cell cycle stage–the corresponding axis (left or right of the matrix) show the range of these values.

Two-dimensional topological skeletons of the mitochondria were manually assigned from the αHSP70 immunofluorescence signal of maximum projected image stacks (Fig. 3a). We evaluated a number of algorithms that potentially allow for automated skeletonisation but were never satisfied with the performance, especially in the recognition of smaller structures. In each individual skeleton we defined the mitochondrial main branch with its orientation from posterior to anterior, junctions, branches, endpoints and loops including their position along the main branch (Fig. 3b). The mitochondrial network size was defined as the sum of the length of all segments of the mitochondrial skeleton in a single cell and ranged from 8.6 to 24.2 μm (Fig. 4a). In order to determine how mitochondrial growth is coordinated with the cell cycle and the cell body size in *T. brucei* BSF, we used the above described refined cell cycle stages and ordered mitochondria by rank according to increasing size (Fig. 4a). For this, each cell was assigned a normalised rank according to the overall mitochondrial network length (Fig. 4b). The cell with the shortest mitochondrion obtained the value 0 and the cell with the longest was assigned the value 1 (Fig. 4b). During the cell cycle the median of the mitochondrial network size increased from 13.8 to 18.5 μm (Fig. 4a). The largest increase was detected during the 2K2N phase of the cell cycle after the mitochondrial genome has been replicated and segregated. We also measured the increase in cell body size during cell cycle using the area as a proxy for overall cell size, which ranged from 18.2 ± 3.4 μm^2^ in G1 to 28.7 ± 4.8 μm^2^ in G2/M (Fig. 4c,d). Assuming an ellipsoid shape with an average diameter of 1 to 1.5 μm in *T. brucei,* this data is in agreement with previous studies where the volume of long slender cells has been estimated to be 28.4 μm^3 ^41. Plotting cell body size versus the mitochondrial network size showed a positive correlation with a R^2^ value of 0.38 (Fig. 4c). When we analysed the normalised progression of mitochondrial network and the cell body size relative to the cell cycle progression we found that cell body size increased faster than the mitochondrial network size (Fig. 4d). While the cell body size increased by almost 50% during G1 of the cell cycle, the mitochondrial network developed very little during this time and showed the majority of size increase during the last quarter of the cell cycle starting in 2K2N cells. Thus cell body growth precedes organelle growth, and mitochondrial growth in *T. brucei* BSF is not continuous throughout the cell cycle as it has been proposed in other Eukaryotes including human cells, budding yeast or *Chlorella*^21^,^22^,^23^. Aside from the network size, we also tested other features including the number of endpoints, junctions, loop perimeter, loop area and loop number for any statistically significant correlation to the cell cycle progression in BSF cells (Fig. 5, for feature definition see Fig. 3b,c). Besides the network size, the number of junctions, endpoints and loop perimeter also showed at least partial correlation with the cell cycle progression (Fig. 5). On the other hand, the loop area and the number of loops did not correlate with the cell cycle progression (Fig. 5).

### Positions of mitochondrial growth

**Figure 6:**
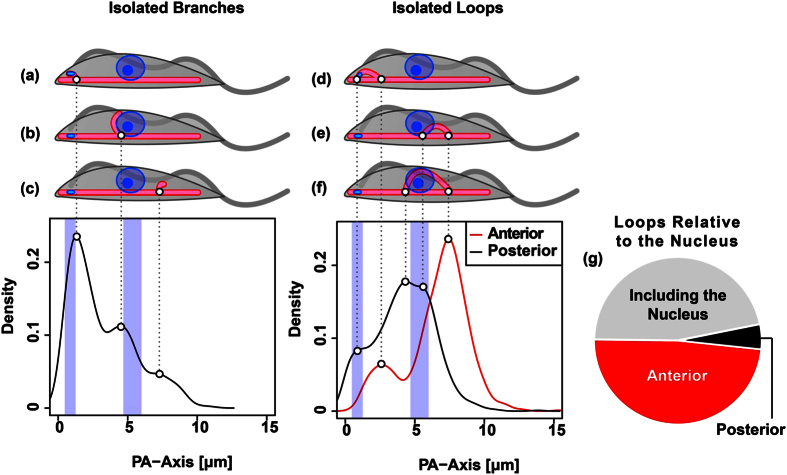
Initial branches and loops. (**a–f**) Depiction of hotspots of mitochondrial outgrowth in early 1K1N cells (n = 901). (**a–c**) Isolated branches can be grouped into three major classes: (**a**) posterior branches that are found close to the kinetoplast, (**b**) branches that are found close to the nuclei but slightly posterior to their centre point, and (**c**) branches emerging from the anterior part of the cell. (**d–f**) Isolated loops. (**d**) Loops on the posterior end of the cell that likely include the kinetoplast, (**e**) loops that are found anterior to the nuclear centre points, (**f**) and loops that include the centre points of the nuclei. Graphs show the calculated kernel density estimators (KDE) of isolated branches (left) and posterior- and anterior junction points emerging from the mitochondrial main branch (right). The blue vertical bars depict the interquartile ranges (IQR) of the projected centre points of the kinetoplasts (left bar) and the nuclei (right bar). (**g**) Pie chart shows manual evaluation of the isolated loops relative to the centroids of the nuclei.

The mitochondrion of BSF trypanosomes consists of the main branch that spans the posterior-anterior axis (PA-axis) of the cell body. This tube, which is distributed asymmetrically in the cell body, mostly close to the plasma membrane opposing the flagellum, is present at any time and outgrowth emanates from this structure. In order to test if outgrowth from the main branch occurs randomly along the PA-axis or if particular sites are preferred, we first analysed the presence of branches and loops along the main branch in 901 early 1K1N cells. For this, the mitochondrial main branch was assigned and mathematically straightened (Fig. S2). The centre of mass of each the nucleus and kDNA was projected onto the main branch according to the minimal Euclidean distance (Fig. S2b,c) and were then used as reference points along the mitochondrial main branch. Loops and branches were assigned to the main branch and the distance from the posterior end of the mitochondrion to the junction or bifurcation points were measured along the mitochondrial main branch. The cumulative data of these junction points was used to calculate kernel density estimators (KDE) (Fig. 6). Peaks in the graphs indicate hotspots of mitochondrial organelle outgrowth. Isolated branches were mainly found in the posterior part of the cell, most prominently either close to the kinetoplast or slightly anterior of the nucleus (Fig. 6a,b). A third less-pronounced hotspot of isolated branches was identified between the nucleus and the anterior tip of the mitochondrion (Fig. 6c). Loops, on the other hand were more frequently detected in the anterior region. For each individual loop we identified the position of both intersections with the main branch and termed them posterior or anterior junctions (Fig. 6d–f). The most frequent intersection with the main branch was an anterior junction, which was located between the nucleus and the anterior tip of the mitochondrion. Two posterior junction points were found to be similar in abundance on either side of the nucleus. Thus the corresponding loops either surround or are found posterior to the nucleus (Fig. 6e,f). These results were also supported by manual evaluation of loops in the cell (Fig. 6g). Another junction-pair of a posterior and an anterior peak building a loop surrounding the kDNA was found in the posterior part of the cell (Fig. 6d).

### Mitochondrial growth patterns during the cell cycle

**Figure 7:**
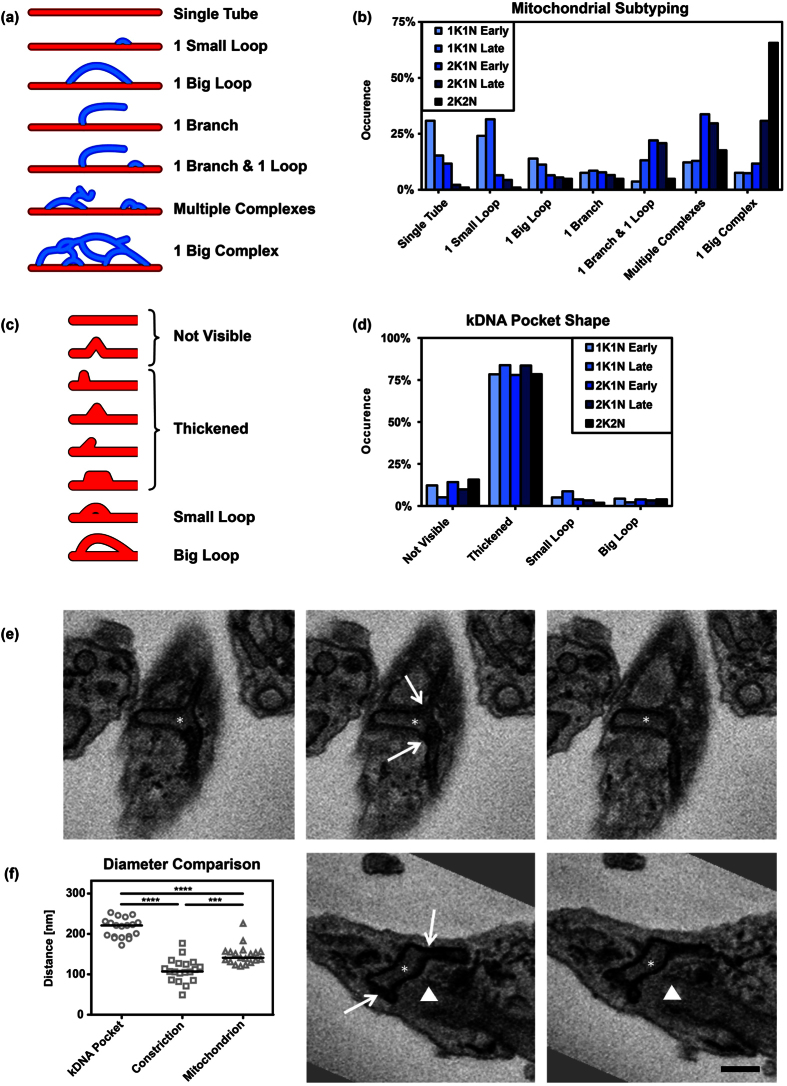
Classification and quantification of mitochondrial morphology subtypes and kDNA. Mitochondria were grouped into seven distinct classes based on their morphology. (**a**) Schematic representation of the subtypes. (**b**) The plots show the relative occurrence within the refined cell cycle stages. (**c**) Different shapes of the mitochondrion in the region of the kDNA. (**d**) Bar plot of the occurrence of the different shapes in the distinct cell cycle stages. (**e**) Serial block face scanning electron microscopy images of BSF trypanosomes. Section of the posterior end of the cell with the kDNA (star) and the basal body (triangle) are shown. Top row shows three successive sections of the kDNA pocket and the constrictions (arrows). Bottom row, two successive sections of the kDNA pocket and the constrictions. (**f**) Quantification of the average maximum diameter of the kDNA pocket (n = 19, 215 nm ± 23.76 nm), the constrictions (n = 19, 110.15 nm ± 29.32 nm), and random positions in the mitochondrion outside the kDNA pocket (n = 19, 147.63 nm ± 24.64 nm). T-test ***p < 0.005, ****p < 0.0005. Scale bar 500 nm.

Based on the absence or presence of branches and loops the mitochondria were grouped into seven subclasses of distinct complexity (Fig. 7a,b) and the subclasses were correlated with the refined cell cycle stages. We found single tubes to be most abundant in early 1K1N cells, while these cells only rarely contained one big- or multiple complex(es). Subclasses with complex mitochondrial morphologies were most frequently found in cells just prior to cell division (2K2N), while these cells only rarely contained a single tube or single tube with just one loop. Thus in general increasing complexity of the organelle was clearly correlated with the cell cycle progression (Fig. 7b).

### Identification of a specialized area around the kDNA–the kDNA pocket

Based on the distribution of mtHSP70 a majority of the observed mitochondria (~75%) showed an enlarged structure in form of a branch or in some orientations of the cell a small loop in the region where the kDNA is localised (Fig. 7c,d). Quantification showed that there is a decrease in HSP70 signal intensity at the position where the DNA stain DAPI shows the strongest intensity (Fig. S3a–e). Thus apparent loop structure might be explained by the lack of mtHSP70 in the kDNA disk. In some imagery there is an increase of mtHSP70 signal in the middle of the kDNA disk, although it does not reach the intensities of the signal outside the kDNA disk area. The reason for the increase of the HSP70 intensity remains unknown (Fig. S3c,e). In order to further investigate this region of the organelle we used serial block face scanning electron microscopy^42^. This technique allowed us to measure the diameter of the mitochondrial organelle around the position of the kDNA relative to random positions along the posterior anterior axis. We analysed 19 cells and found that the average of the largest diameter of the mitochondrion at the kDNA was 215 nm ±24 nm (Fig. 7e,f), while at random positions outside the area of the kDNA the average maximum diameter of the mitochondrial tube was 148 nm ±25 nm (Table S1). Interestingly, between the kDNA pocket and the remaining mitochondrion we found a constriction of the organelle to an average maximum diameter of 110 nm ± 25 nm (Fig. 7e,f) that seems to “separate” the kDNA pocket from the rest of the mitochondrion. We also looked at the orientation of the organelle. While the main branch of the mitochondrion is generally oriented alongside the posterior anterior axis (PA-axis) it changes its orientation in the region of the kDNA. We measured the angle between the PA-axis oriented organelle and its positioning at the kDNA and found it to be tilted by 115° ± 14° (Fig. S4). Using 3D reconstructions from the individual SBFSEM slices we calculated the volume of the elliptically shaped enlarged organelle region to be around 0.07 μm^3^ (Fig. S5). This volume would be sufficient to house the kDNA disc that is around 0.024 μm^3^ based on the assumption of disc like structure with a diameter of 450 nm and a height of about 150 nm in the 1K1N conformation (*T. brucei* kDNA disk size taken from ref. 43). Thus the posterior part of the mitochondrial organelle is enlarged in the region juxtaposed the basal body, tilted relative to the overall orientation of the organelle and constricted on both ends. It forms an elliptical cylinder reminiscent of a golf club head, that houses the mitochondrial genome (kDNA) which, we suggest to name kDNA pocket. The kDNA pocket is also the region of the mitochondrion where the kDNA is replicated^44^ and connected to the base of the flagellum via the tripartite attachment complex (TAC)^34^. The mitochondrial membranes in this region have been shown to resist detergent extraction likely as a consequence of the TAC structure^34^. Thus, the kDNA pocket clearly differs in morphology from the remaining mitochondrial organelle and houses specific functions related to organelle replication and segregation.

### STED microscopy

**Figure 8:**
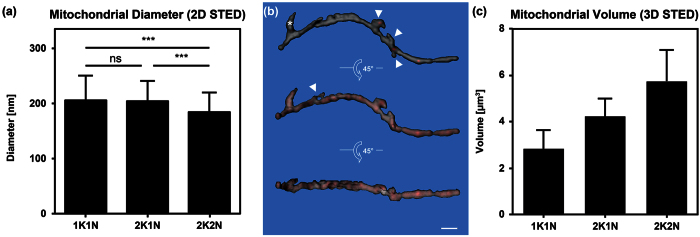
Mitochondrial diameter and size measurements. (**a**) The diameter of BSF trypanosome mitochondria measured by 2D STED: 1K1N cells (n = 102, 205.64 nm ± 45.86 nm), 2K1N cells (n = 104, 204.02 nm ± 36.81 nm) and 2K2N cells (n = 107, 184.29 nm ± 36.17 nm); (ns = p-value > 0.05, ***0.0001 < p-value < 0.001; students’ t-test, two-tailed, unpaired). (**b**) Surface rendering of the mitochondrial volume from a representative 1K1N cell posterior to anterior, left to right. The grey surface is partially transparent to display the maximum intensity projection (red). The 3D reconstruction is turned 45 degrees around the horizontal axis to show the reconstruction in the z axis. Stars mark the kDNA pocket; arrow heads point to mitochondrial branches. Scale bar 1.5 μm. (**c**) Average mitochondrial volumes calculated from 3D reconstructions (1K1N, n = 13, 2.82 μm^3^ ± 0.82 μm^3^; 2K1N, n = 3, 4.21 μm^3^ ± 0.76 μm^3^ and 2K2N n = 3, 5.74 μm^3^ ± 1.35 μm^3^).

In order to achieve better resolution of the mitochondrial shape and size we also applied STED super-resolution microscopy technology that allows a lateral resolution of about 30 nm. Based on these analyses the interquartile range of the diameter of the mitochondrial tubule in the BSF parasites was between 169 nm and 219 nm with an average of 206 nm ± 46 nm (Fig. 8a). This value is larger than the value measured with SBFSEM (148 nm), which might be due to the different resolution of the techniques and or the manual thresholding required in STED imaging. Overall the diameter of *T. brucei* BSF mitochondria appear similar in size to yeast mitochondria from exponentially growing cells analysed by electron microscopy (average diameter 200 nm^45^); however, they seem thinner than mitochondria from stationary yeast cells from the same study that had an average diameter of about 400 nm or mitochondrial organelles isolated from rat liver that showed a mean diameter of 382 nm ±102 nm^46^. More recent life cell imaging of exponentially growing yeast cells by 4Pi-confocal microscopy estimated the organelle diameter to be 339 nm ± 35 nm and 360 nm ± 28 nm in glucose and glycerol containing media, respectively^47^. That the discrepancy between the two studies is caused by fixed vs. life cell imaging or the different imaging techniques (4Pi-confocal vs. transmission electron microscopy) is not clear. Interestingly the diameter of the parasite organelle appeared significantly smaller (p value < 0.001) in 2K2N (G2/M) cells (mean ± s.d. 184 ± 36 nm) when compared to 1K1N (206 ± 46 nm; G1) and 2K1N (204 ± 37 nm; S). The decrease in the diameter thus correlates with the strong increase in network size during the later parts of the cell cycle (Fig. 4d) and one could speculate that stretching of the organelle might contribute to the fast increase in length during G2/M. This is comparable to the exponential vs. stationary growing yeast where during the exponential phase the mitochondrion is thinner than during stationary phase^45^.

In order to evaluate the volume and overall three dimensional morphology of the mitochondrion we acquired images using the 3D STED acquisition mode of the TCS SP8 STED 3X. The resolution after Huygens deconvolution was 70 nm in xy and 100 nm in z, compared to about 30 nm in xy when using 2D STED. We acquired data from 19 cells of which the majority showed the 1K1N confirmation. Using the Imaris software package we reconstructed the 3D volume of the mitochondrial organelle from these cells (see example in Fig. 8b). The reconstructed 3D volumes show a continuous tubular structure with the kDNA pocket and several small branch points along the PA-axis. Compared to confocal or epifluorescence microscopy, where the gaps between the individual z-stacks often become quite evident the 3D STED shows very little distortion (Fig. 8b).

From thirteen 1K1N cells we calculated the average of the mitochondrial volume to be 2.8 μm^3^ ± 0.8 μm^3^ (Fig. 8c, Table S3). In a much smaller number of 2K1N (S) and 2K2N (G2/M) cells we calculated the volume to increase to 4.2 μm^3^ ± 0.8 μm^3^ and 5.7 μm^3^ ± 1.3 μm^3^, respectively. The volume measurements by 3D STED are much larger than the theoretical volumes calculated from the 2D STED or the SBFSEM measurements of the organelle diameter and length. Based on the diameter of the mitochondrial tube measured by 2D STED the organelle should have a volume of 0.4 μm^3^ and 0.6 μm^3^ in 1K1N and 2K2N cells, respectively. We hypothesize that this large difference in volume can be attributed to the loss of resolution especially in the z-axis when using the 3D acquisition mode compared to 2D STED or SBFSEM. The blur then translates during reconstruction into an increased diameter of the organelle mostly in z, but also in xy. If this explanation is true then the 3D STED reconstructed organelle should show a biased diameter distribution with longer diameters in z than in xy. We tested this and measured the average diameter of the tube when viewed from the xy axis and the z axis at more than 20 positions in one example mitochondrion. In xy, the average diameter was 315 nm ± 65 nm, while in z the diameter was 422 ± 115 when using the 3D STED acquisition mode (Table S2). A two-sided unpaired students t-test showed significance of the difference with a p value of p < 0.001. Although the 3D STED reconstructions offer a very large improvement over reconstructions from regular confocal microscopy, the calculations based on these reconstructions likely overestimate the volume of the organelle substantially. In the near future SBFSEM reconstructions will probably solve this issue.

The morphological comparison between mitochondria from BSF trypanosomes and yeast or mammalian organelles have to be viewed in the light of the functional differences of these organelles. Trypanosome mitochondria, for example, only contain one mitochondrial nucleoid per organelle while yeast and mammalian mitochondria harbor tens of nucleoids with different numbers of genomes organized in these structures. Furthermore, the trypanosome BSF organelles almost completely lack the components of the oxidative phosphorylation machinery in the inner mitochondrial membrane and consequently are devoid of cristae whereas the yeast and mammalian organelles contain a large number of cristae even in stationary growth conditions. None the less the comparison indicates the range of mitochondrial organelle morphologies in evolutionary very distant systems.

**Figure 9:**
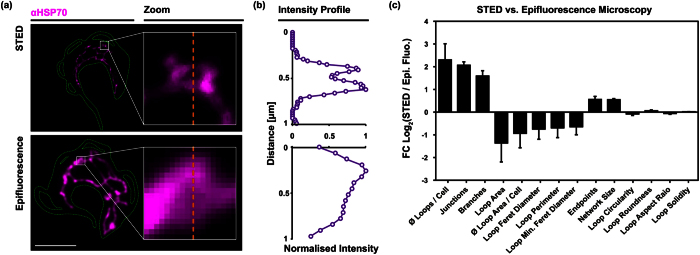
Comparison of epifluorescence microscopy and STED. Comparison of the skeletonised mitochondrial structure based on epifluorescence and super-resolution (STED) microscopic imagery. (**a**) STED resolves small loop structures that are not, or barely visible in epifluorescence microscopy. The magnified regions show two small loops of comparable size and spatial orientation. (**b**) Intensity profiles passing the centre of the loops. The affected pixels are indicated as the orange dashed lines in (**a**). Scale bar 5 μm. (**c**) Log_2_-fold changes of the median ratios (mean ± standard deviation) for each cell cycle stage.

We then compared the median value of several features of the mitochondrial skeletons derived from STED and epifluorescence microscopy (Fig. 9a). The manual comparison of the images revealed that smaller structures, mostly loops, became visible in STED microscopy, while they remained undetected in regular epifluorescence microscopy (Fig. 9a,b). In the measurements from STED images the average number of loops increased by more than fourfold when compared to epifluorescence microscopy (Fig. 9c). Consequently, also the number of junctions and branches increased. On the other hand, the average loop area decreased by approximately twofold. The higher resolution in STED microscopy led to ~50% increase of the overall network length. The apparent increase in size was most clearly seen in the 2K2N population that showed a strong fenestration of the mitochondrial network just prior to cell division (Fig. S6). The strong fenestration is rather surprising since cells in G1 just after cytokinesis basically lack fenestration and instead mostly contain a single mitochondrial tube. This suggests extensive pruning of the organelle just prior or during cell division.

### Model of mitochondrial growth and separation during the cell cycle

**Figure 10:**
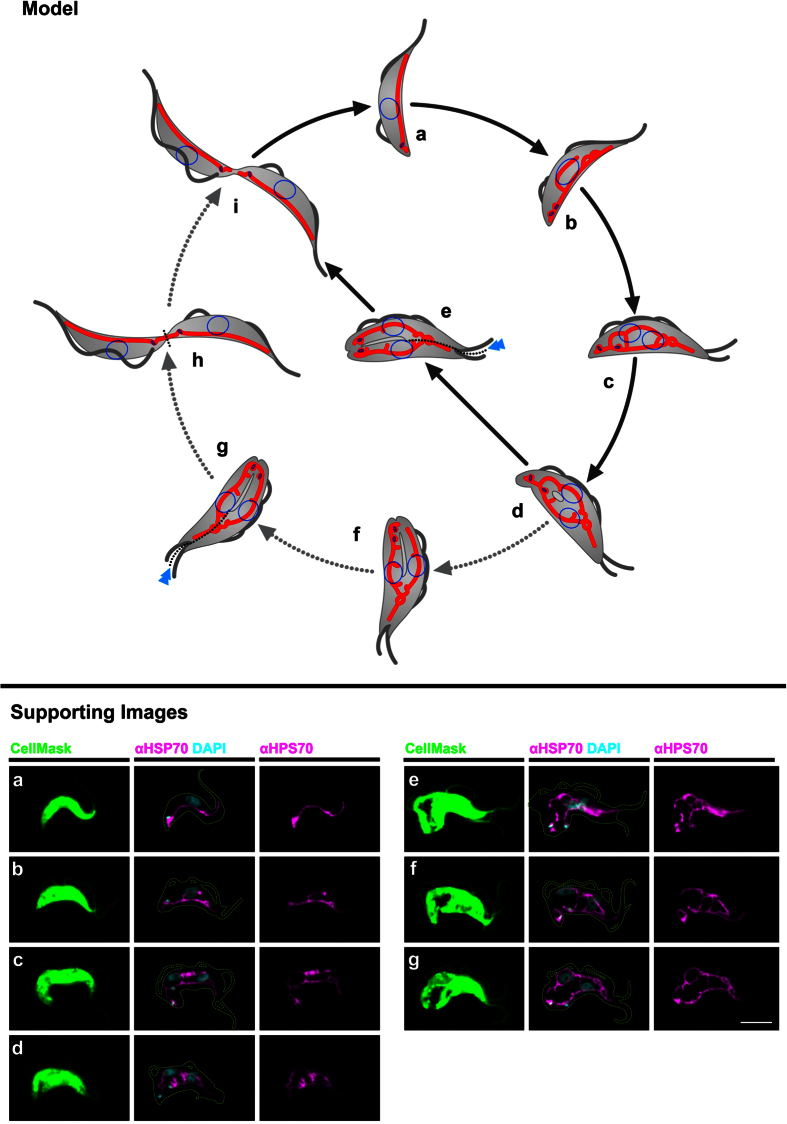
Model of mitochondrial replication and segregation cycle in BSF *T. brucei*. Model (upper part): Model of mitochondrial growth during the cell cycle (**a**-**i**). Cells including the flagella are depicted in gray, nuclei are shown as blue circles inside the cell, kinetoplasts are shown as blue ovals inside the mitochondrion (red). Two alternative pathways are shown; the direct pathway follows the black arrows; the indirect pathway diverts from (**d**) to (**i**) and follows the gray dotted arrows. Supporting images (lower part): representative epifluorescence images (**a–g**) for the support of the model. Green, cell contents stained by CellMask: cyan DNA stained by DAPI and magenta, mitochondrial matrix stained using anti-HSP70 antibodies. Scale bar 5 μm.

Based on our data we propose a model of mitochondrial growth in the BSF parasite that includes growth of a major branch posterior of the nucleus as well as loop formations in the anterior part of the cell (Fig. 10a,b). In the next step, the anterior and posterior network parts fuse to one large network (Fig. 10c). Although fusion events have not been directly demonstrated in *T. brucei,* the expression and subsequent repression of the human pro apoptotic factor Bax strongly suggests that the cells are capable of organelle fusion^31^. We propose two alternative pathways of how the duplicated organelle is separated into two daughter cells after the fused network has formed (Fig. 10e,f). In both pathways the pruning of the network occurs simultaneous with the appearance of the division fold that asymmetrically bisects the two daughter cells^48^. The division fold either leads to (i) fission of the organelle between the two replicated kinetoplasts resulting in two daughter cells with clearly separated kinetoplasts or (ii) the network fission occurs such that the kinetoplasts reside in a connected tubular structure (Fig. 10h). The latter leading to a short stretch of continuous organelle, which in the daughter cell is separated anterior to the kinetoplast. In a subsequent step the organelle is re-fused and then finally separated during or just prior to the abscission process (Fig. 10i). Thus the alternative scenario requires the fusion of the fragmented organelle in the cell containing the new basal body/flagellum and a second fission step to finally separate the organelles in both daughter cells.

Although we have presented evidence for mitochondrial fusion and fission events in *T. brucei,* formal confirmation of these processes in the future will require single cell live imaging techniques that should also reveal how frequently these events occur in the parasite.

## Additional Information

**How to cite this article**: Jakob, M. *et al*. Mitochondrial growth during the cell cycle of *Trypanosoma brucei* bloodstream forms. *Sci. Rep.* **6**, 36565; doi: 10.1038/srep36565 (2016).

## Supplementary Material

Supplementary Information

## References

[b1] McBride H. M., Neuspiel M. & Wasiak S. Mitochondria: More Than Just a Powerhouse. Current Biology 16, R551–R560 (2006). 16860735 10.1016/j.cub.2006.06.054

[b2] Borst P. Mitochondrial nucleic acids. Annu. Rev. Biochem. 41, 333–376 (1972). 4563438 10.1146/annurev.bi.41.070172.002001

[b3] Taanman J.-W. The mitochondrial genome: structure, transcription, translation and replication. Biochimica et Biophysica Acta (BBA)-Bioenergetics 1410, 103–123 (1999). 10076021 10.1016/s0005-2728(98)00161-3

[b4] Nass M. M. & Nass S. Intramitochondrial fibers with dna characteristics. i. fixation and electron staining reactions. The Journal of Cell Biology 19, 593–611 (1963). 14086138 10.1083/jcb.19.3.593 PMC2106331

[b5] Schatz G., Haslbrunner E. & Tuppy H. Deoxyribonucleic acid associated with yeast mitochondria. Biochemical and Biophysical Research Communications 15, 127–132 (1964). 26410904 10.1016/0006-291x(64)90311-0

[b6] Douglas M. G. & Butow R. A. Variant forms of mitochondrial translation products in yeast: evidence for location of determinants on mitochondrial DNA. Proc. Natl. Acad. Sci. USA 73, 1083–1086 (1976). 772678 10.1073/pnas.73.4.1083 PMC430204

[b7] Chacinska A., Koehler C. M., Milenkovic D., Lithgow T. & Pfanner N. Importing mitochondrial proteins: machineries and mechanisms. Cell 138, 628–644 (2009). 19703392 10.1016/j.cell.2009.08.005 PMC4099469

[b8] Westermann B. Mitochondrial fusion and fission in cell life and death. Nature Reviews Molecular Cell Biology 11, 872–884 (2010). 21102612 10.1038/nrm3013

[b9] Youle R. J. & van der Bliek A. M. Mitochondrial fission, fusion, and stress. Science, doi: 10.1126/science.1219855 (2012). PMC4762028 22936770

[b10] Hales K. G. & Fuller M. T. Developmentally Regulated Mitochondrial Fusion Mediated by a Conserved, Novel, Predicted GTPase. Cell 90, 121–129 (1997). 9230308 10.1016/s0092-8674(00)80319-0

[b11] Hermann G. J. . Mitochondrial fusion in yeast requires the transmembrane GTPase Fzo1p. The Journal of Cell Biology 143, 359–373 (1998). 9786948 10.1083/jcb.143.2.359 PMC2132826

[b12] Meeusen S. . Mitochondrial Inner-Membrane Fusion and Crista Maintenance Requires the Dynamin-Related GTPase Mgm1. Cell 127, 383–395 (2006). 17055438 10.1016/j.cell.2006.09.021

[b13] Kornmann B. . An ER-Mitochondria Tethering Complex Revealed by a Synthetic Biology Screen. Science 325, 477–481 (2009). 19556461 10.1126/science.1175088 PMC2933203

[b14] Friedman J. R. . ER tubules mark sites of mitochondrial division. Science 334, 358–362 (2011). 21885730 10.1126/science.1207385 PMC3366560

[b15] Lang A., John Peter A. T. & Kornmann B. ER–mitochondria contact sites in yeast: beyond the myths of ERMES. Current Opinion in Cell Biology 35, 7–12 (2015). 25836730 10.1016/j.ceb.2015.03.002

[b16] Cannino G., Di Liegro C. M. & Rinaldi A. M. Nuclear–mitochondrial interaction. MITOCHONDRION, doi: 10.1016/j.mito.2007.07.001 (2007). 17822963

[b17] Rinaldi A. M. . Biochemical and electron microscopic evidence that cell nucleus negatively controls mitochondrial genomic activity in early sea urchin development. Proc. Natl. Acad. Sci. USA 76, 1916–1920 (1979). 287031 10.1073/pnas.76.4.1916 PMC383503

[b18] Parikh V. S., Morgan M. M., Scott R., Clements L. S. & Butow R. A. The mitochondrial genotype can influence nuclear gene expression in yeast. Science 235, 576–580 (1987). 3027892 10.1126/science.3027892

[b19] Schmidt O., Pfanner N. & Meisinger C. Mitochondrial protein import: from proteomics to functional mechanisms. Nature Reviews Molecular Cell Biology 11, 655–667 (2010). 20729931 10.1038/nrm2959

[b20] Schulz C., Schendzielorz A. & Rehling P. Unlocking the presequence import pathway. Trends in Cell Biology 25, 265–275 (2015). 25542066 10.1016/j.tcb.2014.12.001

[b21] Posakony J. W., England J. M. & Attardi G. Mitochondrial growth and division during the cell cycle in HeLa cells. The Journal of Cell Biology 74, 468–491 (1977). 885911 10.1083/jcb.74.2.468 PMC2110063

[b22] Atkinson A. W. Jr., John P. C. L. & Gunning B. E. S. The growth and division of the single mitochondrion and other organelles during the cell cycle of Chlorella, studied by quantitative stereology and three dimensional reconstruction. Protoplasma 81, 77–109 (1974). 4420697 10.1007/BF02055775

[b23] Tanaka K., Kanbe T. & Kuroiwa T. Three-dimensional behaviour of mitochondria during cell division and germ tube formation in the dimorphic yeast Candida albicans. Journal of Cell Science 73, 207–220 (1985). 3894384 10.1242/jcs.73.1.207

[b24] Vickerman K. The mechanism of cyclical development in trypanosomes of the Trypanosoma brucei sub-group: An hypothesis based on ultrastructural observations. Trans R Soc Trop Med Hyg 56, 487–495 (1962). 13997060 10.1016/0035-9203(62)90072-x

[b25] Vickerman K. Polymorphism and mitochondrial activity in sleeping sickness trypanosomes. Nature 208, 762–766 (1965). 5868887 10.1038/208762a0

[b26] Hajduk S. & Ochsenreiter T. RNA editing in kinetoplastids. RNA Biol 7, 229–236 (2010). 20220308 10.4161/rna.7.2.11393

[b27] Priest J. W. & Hajduk S. L. Developmental regulation of mitochondrial biogenesis in Trypanosoma brucei. J Bioenerg Biomembr 26, 179–191 (1994). 8056785 10.1007/BF00763067

[b28] Tyler K. M., Matthews K. R. & Gull K. Anisomorphic cell division by African trypanosomes. Protist 152, 367–378 (2001). 11822664 10.1078/1434-4610-00074

[b29] Chanez A. L. Ablation of the single dynamin of T. brucei blocks mitochondrial fission and endocytosis and leads to a precise cytokinesis arrest. Journal of Cell Science 119, 2968–2974 (2006). 16787942 10.1242/jcs.03023

[b30] Morgan G. W. The Single Dynamin-like Protein of Trypanosoma brucei Regulates Mitochondrial Division and Is Not Required for Endocytosis. Journal of Biological Chemistry 279, 10692–10701 (2003). 14670954 10.1074/jbc.M312178200

[b31] Esseiva A. C. . Temporal dissection of Bax-induced events leading to fission of the single mitochondrion in Trypanosoma brucei. EMBO Rep. 5, 268–273 (2004). 14968134 10.1038/sj.embor.7400095 PMC1299006

[b32] Steinert M. & Van Assel S. Coordinated replication of nuclear and mitochondrial desoxyribonucleic acids in “Crithidia luciliae”. Arch. Int. Physiol. Biochim. 75, 370–371 (1967). 4166472

[b33] Woodward R. & Gull K. Timing of nuclear and kinetoplast DNA replication and early morphological events in the cell cycle of Trypanosoma brucei. Journal of Cell Science 95(Pt 1), 49–57 (1990). 2190996 10.1242/jcs.95.1.49

[b34] Ogbadoyi E. O. A High-Order Trans-Membrane Structural Linkage Is Responsible for Mitochondrial Genome Positioning and Segregation by Flagellar Basal Bodies in Trypanosomes. Molecular Biology of the Cell 14, 1769–1779 (2003). 12802053 10.1091/mbc.E02-08-0525 PMC165075

[b35] Wirtz E., Leal S., Ochatt C. & Cross G. A. A tightly regulated inducible expression system for conditional gene knock-outs and dominant-negative genetics in Trypanosoma brucei. Molecular & Biochemical Parasitology 99, 89–101 (1999). 10215027 10.1016/s0166-6851(99)00002-x

[b36] Klein K. G., Olson C. L. & Engman D. M. Mitochondrial heat shock protein 70 is distributed throughout the mitochondrion in a dyskinetoplastic mutant of Trypanosoma brucei. Molecular & Biochemical Parasitology 70, 207–209 (1995). 7637705 10.1016/0166-6851(95)00013-q

[b37] Hell S. W. & Wichmann J. Breaking the diffraction resolution limit by stimulated emission: stimulated-emission-depletion fluorescence microscopy. Opt. Lett., OL 19, 780–782 (1994). 10.1364/ol.19.000780 19844443

[b38] Deerinck T. J., Eric A., Andrea T. & Mark E. Ncmir methods for 3d em: a new protocol for preparation of biological specimens for serial blockface scanning electron microscopy. *ncmir.ucsd.edu* Available at: https://www.ncmir.ucsd.edu/sbem-protocol/. (Accessed: 10 August 2016).

[b39] Kremer J. R., Mastronarde D. N. & McIntosh J. R. Computer Visualization of Three-Dimensional Image Data Using IMOD. Journal of Structural Biology 116, 71–76 (1996). 8742726 10.1006/jsbi.1996.0013

[b40] Siegel T. N., Hekstra D. R. & Cross G. A. M. Analysis of the Trypanosoma brucei cell cycle by quantitative DAPI imaging. Molecular & Biochemical Parasitology 160, 171–174 (2008). 18501977 10.1016/j.molbiopara.2008.04.004 PMC2495048

[b41] Hecker H., Burri H., Steiger R. & Geigy R. Morphometric data on the ultrastructure of the pleomorphic bloodforms of Trypanosoma brucei, Plimmer and Bradford, 1899. Acta Tropica 29, 182–198 (1972). 4402810

[b42] Denk W. & Horstmann H. Serial block-face scanning electron microscopy to reconstruct three-dimensional tissue nanostructure. Plos Biol, doi: 10.1371/journal.pbio.0020329 (2004). PMC524270 15514700

[b43] Trikin R. . TAC102 Is a Novel Component of the Mitochondrial Genome Segregation Machinery in Trypanosomes. PLoS Pathog. 12, e1005586 (2016). 27168148 10.1371/journal.ppat.1005586 PMC4864229

[b44] Povelones M. L. Beyond replication: division and segregation of mitochondrial DNA in kinetoplastids. Molecular & Biochemical Parasitology 196, 53–60 (2014). 24704441 10.1016/j.molbiopara.2014.03.008

[b45] Hoffmann H. P. & Avers C. J. Mitochondrion of yeast: ultrastructural evidence for one giant, branched organelle per cell. Science 181, 749–751 (1973). 4579683 10.1126/science.181.4101.749

[b46] Baudhuin P. & Berthet J. Electron microscopic examination of subcellular fractions. II. Quantitative analysis of the mitochondrial population isolated from rat liver. The Journal of Cell Biology 35, 631–648 (1967). 4294244 10.1083/jcb.35.3.631 PMC2107164

[b47] Egner A., Jakobs S. & Hell S. W. Fast 100-nm resolution three-dimensional microscope reveals structural plasticity of mitochondria in live yeast. Proc. Natl. Acad. Sci. USA 99, 3370–3375 (2002). 11904401 10.1073/pnas.052545099 PMC122530

[b48] Wheeler R. J., Scheumann N., Wickstead B., Gull K. & Vaughan S. Cytokinesis in Trypanosoma brucei differs between bloodstream and tsetse trypomastigote forms: implications for microtubule‐based morphogenesis and mutant analysis. Mol. Microbiol. 90, 1339–1355 (2013). 24164479 10.1111/mmi.12436 PMC4159584

