# Supplementary material for: Mitochondrial growth during the cell cycle of Trypanosoma brucei bloodstream forms
Source: Sci Rep. 2016 Nov 22;6:36565. doi: 10.1038/srep36565 (PMC5118809; doi:10.1038/srep36565)
Supplement: Supplementary Information [file srep36565-s1.pdf]

Supplementary Information

Mitochondrial growth during the cell cycle of *Trypanosoma brucei* bloodstream forms

Martin Jakob[1], Anneliese Hoffmann[1,3], Simona Amodeo[1,3], Camille Peitsch[2,3], Benoît Zuber[2], Torsten Ochsenreiter[1*]

[1]Institute of Cell Biology, University of Bern, Switzerland [2]Institute of Anatomy, University of Bern, Switzerland [3]Graduate School for Cellular and Biomedical Sciences, University of Bern, Switzerland
*Correspondence to: torsten.ochsenreiter@izb.unibe.ch

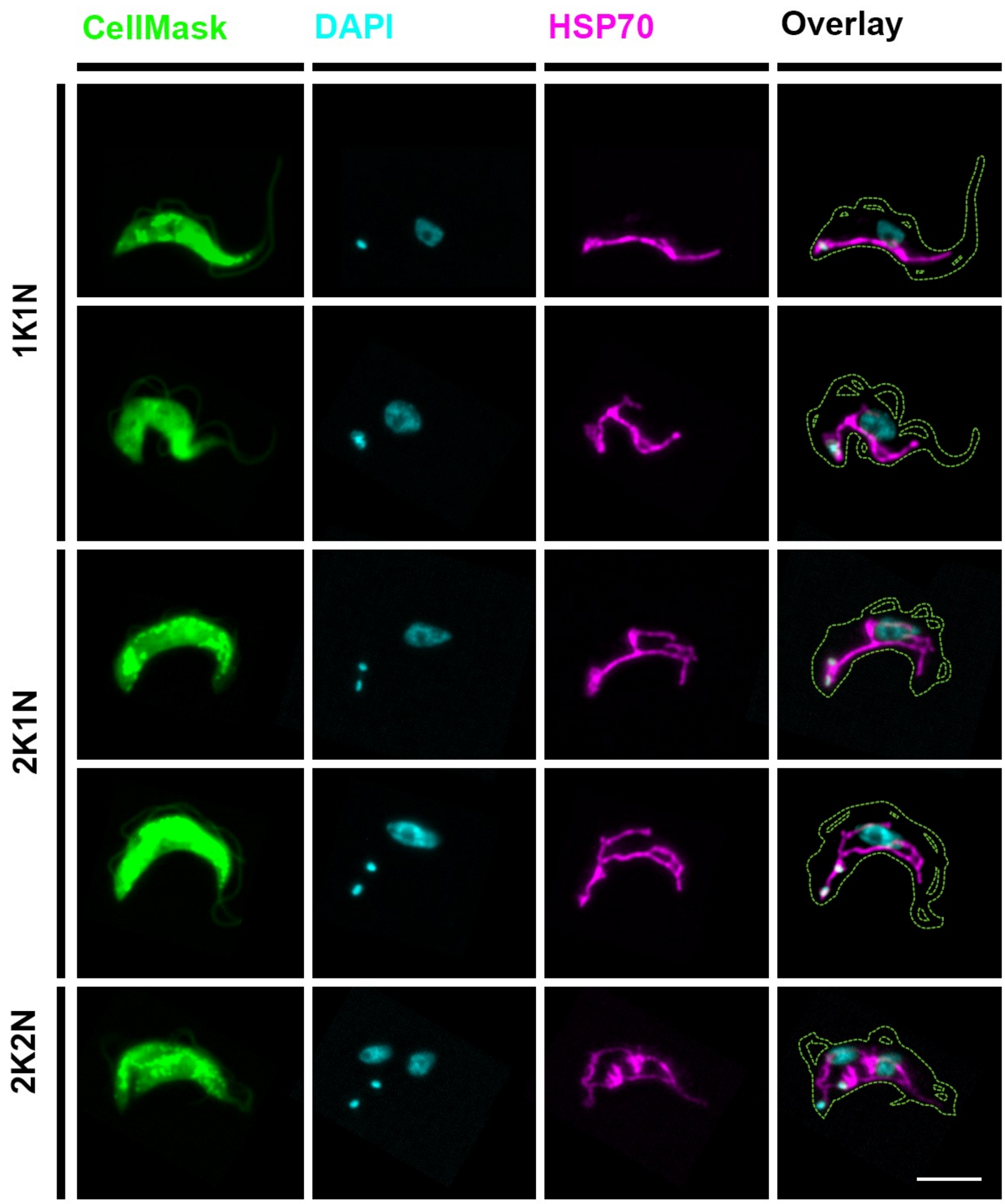

Supplementary Fig. 1 | Epifluorescence microscopy images of BSF *T. brucei* cells stained for the cell body (HSC CellMask), the DNA (DAPI), and the mitochondrion (mtHSP70). Morphologies of the cell body, DNA and the mitochondrial organelle during the cell cycle as observed by epifluorescence microscopy. Green, cell stained by HSC CellMask; cyan, DNA stained by DAPI; and magenta, mitochondrial matrix stained using anti-mtHSP70 antibodies. (Scale bar 5 μm)

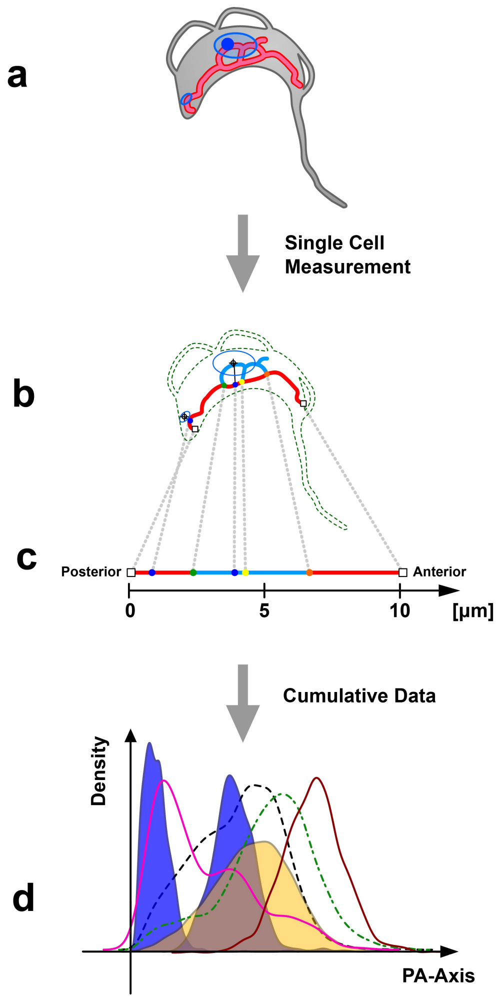

Supplementary Fig. 2 | Schematic representation of the methodology used for the measurements. Images of cells are acquired with an epifluorescent microscope and single cells are recognised. (a) Red depicts the mitochondrion, blue the kDNA and nucleus and grey the cell body and flagellum. (b,c) The main mitochondrial branch is shown in red. Outgrowth is highlighted in blue. The dashed line shows the cell body. (b) A variety of features are extracted for each signal. The centre points of the kinetoplasts and the nuclei are projected onto the mitochondrial main branch according to the shortest Euclidean distance to any point on the main branch. (c) The distances from the posterior tip of the main branch to each event (junction, loop or DNA organelle) are measured. (d) Data from multiple cells are used for the identification of patterns. Absolute distances from the posterior tip to any event are plotted as kernel density estimators (KDE).

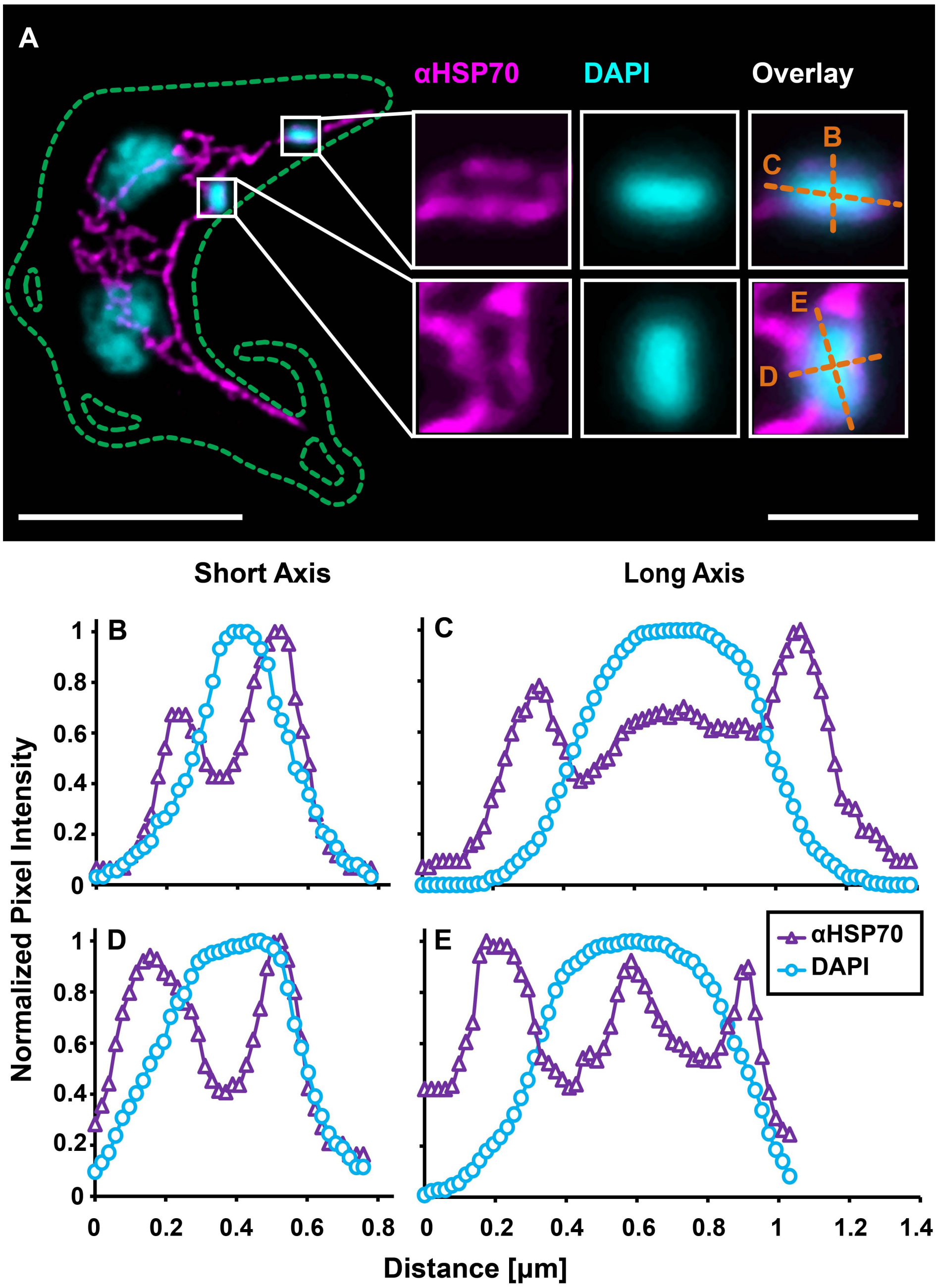

Supplementary Fig. 3 | The presence of the kinetoplast correlates with the lack of mtHSP70 signal. Image of a 2K2N cell. (a) The green, dashed line shows the cell outline; the mtHSP70 signal is shown in magenta; DNA stained by DAPI is shown in cyan. The left part shows the cell - the white squares contain the kinetoplasts. The right part shows a magnification of this region for DNA, mtHSP70 and an their overlay. (Left scale bar 5 µm, right scale bar 1 µm). (b-e) show intensity profiles for DAPI (light-blue) and mtHSP70 (violet). The intensity values are measured as indicated by the dashed, orange lines as shown in (a).

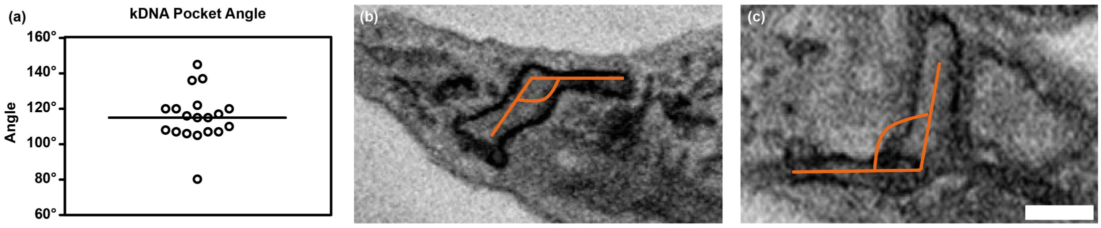

Supplementary Fig. 4: | Orientation of the kDNA pocket relative to the posterior-anterior axis of the mitochondrion. (a) Angle measurements from SBFSEM imagery. (b) Representative imagery. n=19. (Scale bar 500 nm)

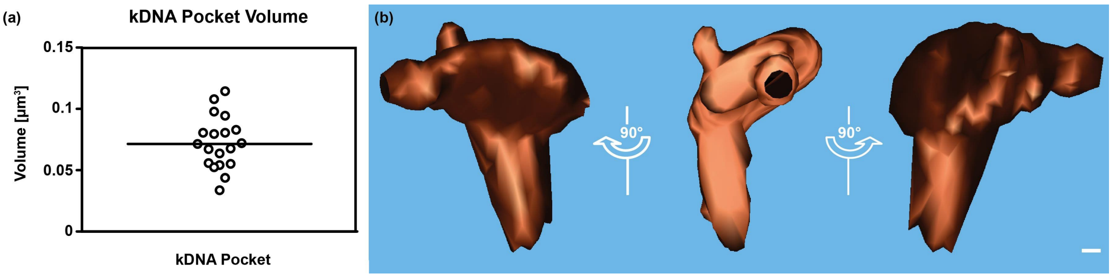

Supplementary Fig. 5 | 3D rendering of the kDNA pocket and volume measurements. (a) kDNA pocket volume calculated from 19 reconstructed pocket regions of BSF trypanosomes. (b) Representative kDNA pocket visualization. Anterior of the cell is on top, posterior towards the bottom. (Scale bar 200 nm)

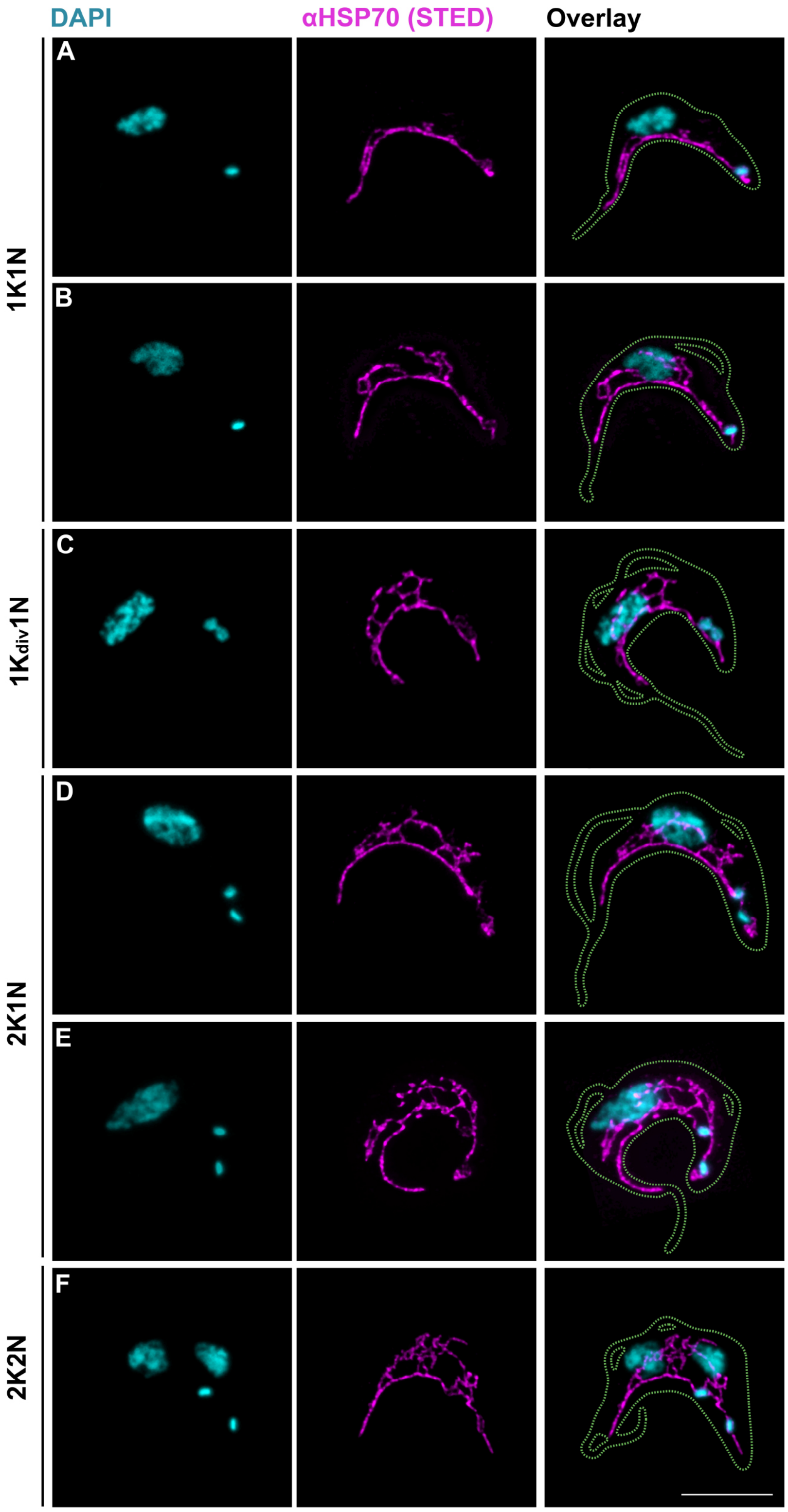

Supplementary Fig. 6 | Mitochondrial morphologies during the cell cycle as observed by STED microscopy. The green, dashed lines depict cell outlines; the mtHSP70 signal is shown in magenta; DAPI is shown in cyan. The rows (a-f) show cells in different cell cycle stages. (Scale bar, 5 µm).

Supplementary Table 1 | kDNA pocket measurements.

	Tube Diameter				
	kDNA Pocket [nm]	Constriction [nm]	Mitochondrion [nm]	Volume [μm^3]	Angle [°]
	201	71	139	0.067	115
	246	49	129	0.108	80
	248	106	120	0.098	105
	194	86	160	0.054	108
	197	103	156	0.055	116
	242	125	123	0.081	117
	228	85	183	0.044	137
	223	114	151	0.08	107
	193	105	123	0.034	122
	221	127	153	0.056	107
	172	107	141	0.094	110
	219	106	135	0.081	106
	190	155	155	0.052	120
	253	130	136	0.114	115
	188	118	137	0.083	120
	227	135	131	0.071	136
	231	82	157	0.068	120
	191	112	150	0.064	107
	221	177	226	0.072	145
Median	221	107	141	0.071	115
Q25	193.5	94.5	133	0.056	107
Q75	229.5	126	155.5	0.082	120
Average	215	110.158	147.632	0.072	115.421
SD	23.767	29.324	24.642	0.021	14.187

F-Test	
kDNA Pocket vs Constriction	0.381056777
Mitochondrion vs Constriction	0.467811584
kDNA Pocket vs Mitochondrion	0.879778634

T-Test

	p-Value	Significance Level
kDNA Pocket vs Constriction	0.0000000000	****
Mitochondrion vs Constriction	0.0001445450	***
kDNA Pocket vs Mitochondrion	0.0000000003	****

Supplementary Table 2 | Mitochondrial diameter from 2D STED imagery

	Mito Diameter (XY) [µm]	Mito Diameter (Z) [µm]
	0.247	0.656
	0.35	0.511
	0.372	0.444
	0.327	0.387
	0.307	0.363
	0.24	0.385
	0.161	0.706
	0.277	0.369
	0.284	0.188
	0.313	0.296
	0.386	0.538
	0.422	0.467
	0.367	0.376
	0.339	0.43
	0.331	0.445
	0.337	0.528
	0.197	0.412
	0.31	0.385
	0.352	0.263
	0.508	0.333
	0.362	0.417
	0.304	0.467
	0.319	0.343
	0.321	
	0.279	
	0.323	
	0.356	
	0.261	
	0.214	
	0.328	
	0.283	
	0.309	
Average	0.315	0.422
SD	0.066	0.116
Q25	0.282	0.366
Median	0.32	0.412
Q75	0.351	0.467

Supplementary Table 3 | Mitochondrial volume calculations from 3D STED reconstructions

	1K1N			2K1N			2K2N		
	Int. [AU]	Area [μm^2]	Vol. [μm^3]	Int. [AU]	Area [μm^2]	Vol. [μm^3]	Int. [AU]	Area [μm^2]	Vol. [μm^3]
	6952	21.1	2.26	1440	32.9	3.83	2378	63.1	7.21
	3265	19.3	2.18	3134	33.3	3.72	2637	44.6	4.56
	5233	14.6	1.58	2364	44	5.1	1383	46.3	5.46
	3477	21.6	2.32						
	4131	31	3.6						
	2844	26.4	3.21						
	3063	19.8	2.02						
	4503	24.6	2.59						
	1499	33.2	4.06						
	1732	21.4	2.13						
	1803	32.2	3.92						
	3342	24.7	3.15						
	2351	28.8	3.74						
Average	3399.615	24.515	2.828	2312.667	36.733	4.217	2132.667	51.333	5.743
SD	1533.64	5.598	0.824	848.166	6.296	0.767	662.02	10.226	1.348
Q25	2351	21.1	2.18	1902	33.1	3.775	1880.5	45.45	5.01
Median	3265	24.6	2.59	2364	33.3	3.83	2378	46.3	5.46
Q75	4131	28.8	3.6	2749	38.65	4.465	2507.5	54.7	6.335
